# Group based metacognitive therapy for alcohol use disorder: a pilot study

**DOI:** 10.3389/fpsyt.2024.1375960

**Published:** 2024-06-28

**Authors:** Julia Kroener, Maja Lara Eickholt, Zrinka Sosic-Vasic

**Affiliations:** ^1^ Department of Applied Psychotherapy and Psychiatry, Christophsbad Goeppingen, Goeppingen, Germany; ^2^ Medical Department, University of Ulm, Ulm, Germany

**Keywords:** alcohol use disorder, AUD, metacognitive training, MCT, group therapy, metacognitions, short-intervention

## Abstract

**Introduction:**

Alcohol use disorder (AUD) is a severe clinical disorder, which has been associated with 5.3% of death worldwide. Although several treatments have been developed to improve AUD symptomatology, treatment effects were moderate, with a certain amount of patients displaying symptom deterioration after treatment termination. Moreover, outpatient treatment placements become increasingly scarce, thus necessitating more efficient treatment options. Therefore, the aim of the present study was to investigate the efficacy, feasibility, and acceptability of a newly invented, short, group based metacognitive therapy (MCT) for patients diagnosed with AUD.

**Method:**

Seven patients were treated with eight sessions of group based MCT using a single case series design with an A-B replication across patients. Patients were assessed one month and one week before treatment, as well as one week and three months after treatment termination.

**Results:**

Patients improved significantly and with large effect sizes regarding dysfunctional metacognitive beliefs, desire thinking/craving and depressive symptoms up to three months after treatment termination. AUD symptomatology as well as positive and negative metacognitive beliefs improved at post-treatment, but improvements could not be maintained at follow-up. All included patients completed the treatment and were highly satisfied.

**Conclusion:**

The presented findings show preliminary evidence for the efficacy, feasibility, and acceptability of the implemented group based MCT treatment. Large scale randomized controlled trials (RCTs) are needed to confirm the effectiveness of the developed program for patients diagnosed with AUD.

## 1 Introduction

Alcohol Use Disorder (AUD) is characterized by an inability to regulate alcohol consumption, a compelling craving to consume alcohol, and a continued consumption resulting in interpersonal difficulties as well as an inability to fulfill important role obligations (DSM-5, 1). The detrimental consumption of alcohol is a globally well-known health hazard that has been linked to 5.1% of global burden of disease, and 5.3% of all deaths worldwide (2). Moreover, nocuous alcohol consumption has been linked to various mental health difficulties, including suicide (3–5), heightened susceptibility to major depression and anxiety (6, 7), instances of domestic violence and child abuse (8, 9), and increased rates of workplace absenteeism (10).

Numerous therapeutic concepts have been devised to provide theoretical frameworks and targeted treatments. For example, cognitive behavioral models have emphasized the significance of underlying cognitive biases (11), dysfunctional cognitive beliefs (12) learning processes (13, 14), as well as expectations that sustain the use of alcohol as a coping mechanism for managing negative emotions (15) or achieving desired objectives in both the development and perpetuation of AUD. Cognitive-behavioral therapy (CBT) endeavors to diminish the potent reinforcing influences of alcohol through several strategies, such as conducting situational analysis, developing adequate coping skills and problem management (e.g., refusal training, emotion regulation skills), and increasing alternative activities (for a review see 16). Despite CBT´s significant contributions to the management of AUD, it is important to acknowledge that this approach is not exempt from certain limitations. The moderate effectiveness of CBT in treating AUD in comparison to other treatment approaches, such as medical management or active psychosocial treatments, may be attributed to several structural weaknesses (17–20).

Based on the Self-Regulatory Executive Functioning (S-REF) model proposed by Wells and Matthews (21), scholars have posited that the limited efficacy of cognitive-behavioral therapy (CBT) might be attributed to the persistence of residual symptoms and mechanisms on the metacognitive level (22, 23). While metacognition refers to the cognitive awareness and understanding of one’s own thinking (24), CBT primarily focuses on modifying biased cognitive beliefs, such as the assumption that alcohol consumption is necessary to cope with a problem. However, it is important to note that this alteration does not directly impact metacognitive beliefs, which are believed to drive maladaptive cognitive processes such as worry, rumination, and desired thinking according to the S-REF model (for an overview see 25). According to Spada et al. (23), metacognitive beliefs can be categorized into three subgroups: (1) General metacognitive beliefs, which are related to internal cognitive-affective experiences and their attributed meaningfulness (e.g. “I need to be able to constantly control my thought process”); (2) Positive metacognitive beliefs regarding the effectiveness of cognitive-affective strategies (e.g. “Worrying will help me prepare”) that are associated with the activation of the CAS; and (3) Negative metacognitive beliefs regarding the controllability and risk of mental events (e.g. “I cannot control my thoughts about alcohol”). Moreover, various studies have shown that the described metacognitive beliefs contribute to the activation as well as maintenance of psychiatric symptoms across several clinical disorders, such as affective disorders (26–28), addictive behaviors (29, 30), eating disorders (31, 32), schizophrenia (33), and personality disorders (34), as well as to the maintenance of specific transdiagnostic symptoms, such as emotion dysregulation (32, 35, 36). The utilization of the S-REF model has given rise to a new and innovative approach in the field of psychological therapy known as Metacognitive Therapy (MCT; 37). Within MCT, psychological problems are believed to be sustained by the activation of a mechanism called Cognitive-Attentional Syndrome (CAS), which becomes enabled during times of heightened distress. The CAS constitutes of a variety of dysfunctional cognitive processes such as thought suppression, recurrent negative thinking (e.g., rumination), avoidance, and maladaptive self-monitoring. Once the CAS becomes activated, a heightened attentional emphasis towards distress congruent information will follow. This, in turn, leads to a feedback loop that is ineffective in regulating threatening maladaptive thoughts (for a thorough introduction on MCT and CAS see 37).

Past studies have shown evidence that AUD can be conceptualized from a metacognitive standpoint (e.g., 23, 38–40). Specifically, Spada et al., (39) propose a triphasic metacognitive model of problem drinking. Within the first stage, called the pre-alcohol use phase, alcohol-related cues, such as memories, thoughts, mental images, or alcohol-related cravings are being activated, resulting in the activation of positive metacognitive beliefs regarding alcohol consumption, which in turn result in perseverative thinking styles, like, for example, rumination, desire thinking, and worry. These perseverative thinking styles then result in an increase in craving as well as aversive, negative emotions, therefore reinforcing negative metacognitive beliefs about the necessity to control ones thoughts, increasing the probability of alcohol consumption. During the second stage, called the alcohol use phase, positive metacognitive beliefs about alcohol use are being activated, concurrent with a decrease in metacognitive monitoring, leading to dysregulated drinking. Across this time-period, while the alcohol consumption increases in severity, negative metacognitive beliefs about the inability to control ones alcohol intake, as well as alcohol-related thoughts develop, further contributing to the maintenance of uncontrolled alcohol consumption. During the final stage, the post-alcohol use phase, positive metacognitive beliefs about ruminating about the binge drinking episode are being activated, including the worrying about the emotional, cognitive, and physical effects of uncontrolled alcohol consumption. Paradoxically, this thought process in turn results in a rise in negative affect as well as alcohol-related thoughts, increasing metacognitive beliefs about the latter thoughts. Lastly, in order to suppress those thoughts and to regulate the associated negative emotions, alcohol is being consumed as a dysfunctional coping mechanism, resulting in the maintenance of AUD. This theory is being supported by scientific findings on metacognitive beliefs: For example, metacognitive beliefs have been proposed as a factor that triggers the activation of AUD related components within the CAS, such as monitoring for external and internal alcohol related cues, recurrent intrusive thoughts about alcohol, as well as decreased adaptive metacognitive monitoring (e.g., 41). Moreover, CAS inherent cognitive processes, such as rumination, desire thinking, and worry have been found to be strongly linked to craving and alcohol intake in healthy as well as clinical populations (42–47). For example, a path analysis conducted by Janssen (48) revealed that positive metacognitions about alcohol have a direct impact on both alcohol consumption and desire thinking. Desire thinking, in turn, further increases the likelihood of alcohol consumption, as a conscious, cognitive process of creating positive retrospective as well as prospective mental images about alcohol consumption (e.g., creating mental images about how much fun it was to drink last night), as well as positive self-verbalization about worthwhile reasons to consume alcohol (e.g., alcohol will help me relax and feel good) is being initiated (49). Furthermore, previous studies have shown a strong connection between metacognitive beliefs and various forms of perseverative and repetitive thinking, specifically within the context of AUD (41, 50, 51). For example, a study by Spada et al. (22) has demonstrated that in a sample of individuals with problematic drinking habits, cognitive beliefs about regulating and controlling ones alcohol related cognitions are predictive of alcohol usage and relapse for up to 12 months after treatment termination. Additionally, findings by Spada and Wells (52) have revealed that metacognitive beliefs tend to be increased among individuals with problem drinking. Moreover, Hamonniere et al. (41) demonstrated that repetitive thinking is predictive of AUD severity depending on gender, beliefs about controllability of ones thoughts, as well as metacognitive beliefs.

Interestingly, while there is substantial evidence for the efficacy of MCT in treating several clinical disorders, such as depression, generalized anxiety disorder, post-traumatic stress disorder, or schizophrenia (for a meta-analysis see 53, 54), comparably little research has focused on MCT for AUD. Solely one study conducted by Caselli et al. (55) has shown that treating five patients diagnosed with AUD by implementing 12 sessions of individual MCT can significantly reduce alcohol use and binge drinking, as well as metacognitive beliefs. Given the considerable demand for outpatient therapy among individuals diagnosed with AUD, individual therapy may not be adequate to fulfill this increasing need for treatment. Therefore, the current study aims to extend upon prior research on MCT for AUD by examining the efficacy and feasibility of a brief group based MCT intervention for individuals who have been diagnosed with AUD. More precisely, we investigated whether the implemented short-intervention is an efficient method to reduce dysfunctional metacognitive beliefs, desired thinking, as well as AUD related symptoms, and depressive symptoms. We did not specifically focus on alcohol abstinence, as this has been frequently reported as an obstacle when maintaining patient engagement Connor et al. (56). Rather, we focused on monitoring for a controlled drinking objective, as within the metacognitive framework, it can be argued that actively pursuing a controlled drinking objective is more likely to improve metacognitive control compared to abstaining from alcohol. Moreover, we implemented a standardized group setting, as available therapeutic outpatient placements become increasingly scarce, therefore necessitating a more economical approach to deliver indispensable therapeutic programs to this underserved population.

## 2 Materials and methods

### 2.1 Design

The study utilized a single case series design with an A-B replication across patients, incorporating follow-up measures (57). Patients were allocated to a baseline period of three weeks without receiving any treatment. This procedure was implemented to establish individual baselines that may serve as control periods. There were four measurement time-points across the study (T0 = 4 weeks before first group therapy session, T1 = one week before first group therapy session, T2 = one week after last group therapy session, and T3 = three months after last group therapy session. Patients received reminders per e-mail to complete questionnaires, and were able to complete all assessments online via SoSciSurvey.

### 2.2 Participants

Seven patients diagnosed with AUD (3 females & 4 males) were included within this study. The mean age of the patients was 49 years (*SD* = 7.7; see **Table 1** for patient characteristics). The majority of subjects (*N* = 6) were recruited via the substance abuse unit of the Christophsbad Clinic located in Goeppingen (Germany). One patient was recruited via the addiction counselling center. Recruitment took place between May and July 2023. Patients were briefly screened for inclusion and exclusion criteria and invited for a diagnostic interview thereinafter. At the diagnostic interview, patients received general information about the study and the study setting, and provided written informed consent. Afterwards, the M.I.N.I. International Neuropsychiatric Interview (58) was conducted to assess psychiatric comorbidity. Furthermore, the sections E and B of the SCID-I interview (59) were implemented to assess for symptoms of alcohol dependence, as well as for other addictive disorders, and to except acute psychotic symptoms. The Beck Suicidal Ideation Scale was used to assess suicidality. The study was conducted in accordance with the Declaration of Helsinki, and approved by the Ethics Committee of the Medical Board Baden-Wuerttemberg.

**Table 1:** Patient characteristics.

	Patient 1	Patient 2	Patient 3	Patient 4	Patient 5	Patient 6	Patient 7
Age	50–55	55–60	40–45	55–60	55–60	40–45	45–50
Sex	male	female	male	male	female	male	female
Marital status	married (living separately)	divorced	married	married	divorced	single	single
Education level	University degree	University degree	Completed apprenticeship	Completed apprenticeship	Completed apprenticeship	Secondary school certificate	Secondary school certificate
Duration & abuse history	Addiction started in 2013. Sharp increase in alcohol consumption since 2018, up to 4 bottles of hard liquor (0.7l each) per day; managed to stay abstinent for approx. 6 weeks in 2021, 2022 and 2023	Increased consumption during adolescence and young adulthood. Between 20 and 40 years of age unobtrusive consumption. Consumption increase at age 45; in the last 5 years approx. 1–3 bottles of wine per day; she managed to stay abstinent for 3 month in 2019, afterwards she only managed to stay abstinent on individual days	Addiction started at the age of 26 years. Increased consumption for 10 years, recent consumption 8 bottles of beer and several cans of Jack Daniels per day. Repeatedly managed to stay abstinent for 1–3 months per year	Addiction started 1989; abstinent for several years, he started drinking again in 2021 (daily consumption of beer and hard liquor). Since November 2021 completely abstinent.	From 1992 daily consumption until 2005, then 9 years of abstinence; Since 2014 increased consumption (especially beer and wine); on some days she was able to remain abstinent;	Multiple substance use in the past. He achieved abstinence from alcohol and drugs when he started substitution treatment in 2011 until 2016. Increased alcohol consumption after 2016. 6 months before treatment: approx. 1 bottle of whisky per day	Increased consumption with 19 years. Addiction started with the age of 26. repeatedly 4 months abstinence Increased consumption since 5–6 years (especially with wine and hard liquor). she had managed to be abstinent for 4 months through therapy
Comorbid diagnoses	Recurrent depressive disorder	Recurrent depressive disorder, reports previous diagnoses of Borderline Personality Disorder	None	Recurrent depressive disorder, chronic pain disorder	Recurrent depressive disorder, post-traumatic-stress disorder, past drug dependence	Past drug dependence; recurrent depressive disorder	Recurrent depressive disorder, post-traumatic stress disorder, past drug dependence
Psychotherapy (in the past)	Multiple inpatient treatments, especially due to withdrawal complications	Multiple inpatient stays, especially complex withdrawal	Multiple inpatient stays, especially complex withdrawal	Multiple inpatient stays, especially complex withdrawal	Multiple inpatient stays, especially complex withdrawal; complex psychosomatic treatment over at least 3 months	Multiple inpatient stays, especially complex withdrawal	Multiple inpatient stays, especially complex withdrawal; complex psychosomatic treatment over at least 3 months
Current Psychopharmacological treatment	Escitalopram	Ramipril & Escitalopram	none	Agomelatin	Buproprion	Quetiapin	none
Reasons/Goals for therapy	Understanding oneself; change patterns; permanent abstinence	Understanding oneself; permanent abstinence	Use time wisely; permanent abstinence	Better handling negative thoughts; focus on what really matters in life, live more freely	Try everything to get away from alcohol; more self-confidence; permanent abstinence	Permanent abstinence, wants to turn life around	New perspective; become calmer, better focus on the essential things in life
Number of Completed Treatment Sessions	7	7	8	8	7	7	6

Inclusion criteria were: (a) diagnosis of AUD according to the SCID-I interview (59), and (b) minimum age of 18 years. Exclusion criteria were: (a) acute psychotic symptoms; (b) bipolar disorder; (c) consumption of substances other than alcohol and nicotine during the last three months, (d) concurrent psychological treatment; (e) severe cognitive deficits, (f) acute suicidality or self-harm, and (g) lack of German proficiency. Moreover patients were excluded if they missed more than two group sessions (> 25%) during treatment.

### 2.3 Measures

#### 2.3.1 Meta-cognitions questionnaire 

The MCQ-30 (60) is a 30-item self-report scale assessing five dimensions of metacognitive beliefs: (1) positive beliefs about worry, which assess the propensity to preservative thinking, (2) negative beliefs about the uncontrollability and dangerousness of thoughts, (3) cognitive confidence in oneself, which assesses the degree of confidence in one’s ability to remember and pay attention, (4) beliefs about the need to control ones thoughts, and (5) cognitive self-confidence, which assesses the supervision/monitoring of thought processes. Moreover, an overall score can be built. Each item can be rated on a 4-point Likert-scale ranging from *1* (“do not agree”) to *4* (“agree very much). The internal consistency of the questionnaire was good, with a Cronbach’s α = .84.

#### 2.3.2 Positive and negative alcohol metacognition scale 

Through the PAMS part of the survey, positive beliefs about the need to consume alcohol as a self-regulatory strategy (metacognitive beliefs) are measured. The NAMS part in the questionnaire assesses negative metacognitive beliefs about the uncontrollability and cognitive harm of alcohol use (61). Items are answered on a four-point Likert scale (*1* = “I strongly disagree” to *4* = “I strongly agree.”). Moreover, an overall score can be built. The internal consistency with in this sample was good, with Cronbach’s alpha ranging between.71 -.83.

#### 2.3.3 Desire thinking questionnaire 

The DTQ (62) assesses “desire thinking/craving thoughts.” It consists of ten items divided into two factors. Five items are assigned to “verbal preservation” and five items to “imaginal prefiguration”. Each item consists of a statement describing elaborative thoughts about desired alcohol consumption (e.g., “I mentally repeat to myself that I need to drink.” Or “I imagine how I would feel if I drank alcohol.”). Respondents are asked to estimate how often they use such thinking patterns. The internal consistency of the questionnaire was excellent (Cronbach’s α = .95).

#### 2.3.4 Beck depression inventory

The BDI-II (63) was used to measure depressive symptoms. The self-report questionnaire assesses depression severity at hand of 21 items. Subjective scoring is based on a 4-item choice matrix, with items rated with 0 indicating no clinical symptomatology, and items rated with 3 indicating severe clinical symptoms. The internal consistency of the questionnaire is good (Cronbach’s α = .88).

#### 2.3.5 Alcohol use disorder identification test

Alcohol use, as well as associated consequences, were assessed using the AUDIT (64). Within the self-report questionnaire, harmful or high-risk alcohol usage, as well as fully developed dependence can be evaluated. The instrument consists of 10 questions about alcohol use, covering three domains: hazardous alcohol use, harmful of use, and symptoms of dependence. The internal consistency of the questionnaire in this sample was excellent, with a Cronbach’s α = .91.

#### 2.3.6 Patient satisfaction questionnaire (self-developed)

At T3, patient satisfaction was assessed using a self-developed questionnaire. A specially designed questionnaire was used for this purpose. The aim was to cover as many aspects of patient satisfaction as possible. The questionnaire contained 20 items, as well as the possibility to give free text feedback on the group programme. Examples of items were: “How would you rate the quality of the group programme?” or “How well did the group programme help you to find an appropriate way of dealing with your problems? Patients could choose on a four-point Likert scale (0=bad to 4=very good/excellent or 0=clearly not to 4=clearly yes).

### 2.4 Intervention

The intervention consisted of eight therapeutic sessions implementing metacognitive training within a group setting (see **Table 2** for session content). The duration of each session was 100 minutes (including a 10-minute break). The metacognitive training was delivered according to the basic metacognitive therapy developed by Wells (65) and adapted to treat patients diagnosed with AUD. Furthermore, the metacognitive formulations for alcohol dependence were developed based on Caselli et al. (55).

**Table 2:** Therapy Session Content.

Session	Contents
1	Introduction to MCT, psychoeducation on alcohol dependence, and introduction to the Attention Training Technique (ATT).
2	Introduction to the metacognitive model of alcohol dependence. Creating an individual metacognitive model of alcohol dependence.
3	The Cognitive Attentional Syndrome; referred to in the training as “problem strategies” (rumination, worry, threat monitoring, and thought suppression) and viewed from a metacognitive perspective.
4	Craving/desire for alcohol and how to deal with it
5	Alcohol-specific metacognitions and how to deal with them
6	Thinking biasses and their metacognitive properties
7	Self-esteem in alcohol dependence
8	Relapse prevention

The group sessions were planned to take place twice a week for the first three weeks, and once a week for the remaining two weeks. Due to scheduling difficulties (two patients were unavailable for the first appointment), we were unable to adhere to the planned structure for the first week. Therefore, during the first week, training took place solely once, while the sessions took place twice a week in weeks two to four, and once during week five.

### 2.5 Description of the sessions

A total of eight group sessions were held over a period of five weeks. At the beginning of each session, each patient´s current mood and general mental state was evaluated. During this time-period, patients were able to report any occurring relapses which were subsequently discussed and integrated within the metacognitive model of alcohol dependence (e.g., 30). Afterwards, homework assigned during the previous session was presented by each patient and experienced difficulties were evaluated within the group. Thereinafter, the content for the current session was presented. Each session included a specific ATT exercise (5–12 minutes), which was conducted after reviewing the content for today´s session. Within the second group session, detached mindfulness was introduced, whereas subsequent sessions always included a mindfulness exercise. This mindfulness exercise was continuously incorporated into a session-specific exercise (“the self as an observer” or “postponing brooding”). The concept of ATT as well as Detached Mindfulness and associated effects were incorporated within the metacognitive model of AUD. Practical exercises were conducted for each of the psychoeducational topics covered within the sessions, and homework assignments were given to foster transfer to everyday life. At the end of each session, the homework assignment was reviewed, and patients had the opportunity to provide feedback on today´s group session. Finally, patients received written information about today´s session´s content to take home.

### 2.6 Statistical analysis

The statistical programme SPSS 29 (66) was used to analyse the data.

According to the implemented single case series design, data was visually inspected per patient using frequency distributions, histograms, means, and standard deviations, in order to determine treatment effects. This procedure allows for the evaluation of each individual´s change over time, as well as the assessment of each patient´s range and stability of change. However, the mere evaluation of descriptive data might result in Type I error. Henceforth, changes in outcome measures were analyzed using percentage values.

Moreover, paired sample *t*-tests across all measurement time-points (i.e., T0, T1, T2, T3) were implemented to assess changes from within the waiting period (T0-T1), as well as from pre-treatment (T1) to post-treatment (T2) and follow-up (T3) for the overall group. Pre-, to post and follow-up effect sizes were calculated using Cohen´s *d* (1988).

## 3 Results

### 3.1 Changes in alcohol abuse symptoms, craving, and depression

**Table 3** reports scores for all patients, measurements and measurement time-points. Looking at alcohol abuse symptomatology (AUDIT), symptoms did not change during the control period (T0-T1), *t*(6) = 0.50, *p* ≤.05, *d* = 0.19, however, there was a trend 13% reduction of alcohol abuse symptomatology one week after treatment termination, *t*(6) = 1.52, *p* = .09, *d* = 0.57. No change was observed from pre-treatment to follow-up, *t*(5) = 0.99, *p* = .18, *d* = 0.40 for the group as a whole. Looking at individual scores, patients 3, 4, and 6 did not consume alcohol during the entire assessment period (i.e., T0-T3). Additionally, patient 2 consumed alcohol solely once with one glass of wine at T2. Patients 1 and 7 did show a decrease in the number of times they consumed alcohol (from 2–4 times a month to once a month), however, the amount of drinks consumed did not change. Lastly, patient 5 showed an increase in the number of times she consumed alcohol (from once a month to 2–4 times a month), however, the amount of drinks consumed each time decreased from 5–6 drinks to 1–2.

**Table 3:** Outcome measures and symptom reduction across MCT group therapy.

		Patient 1	Patient 2	Patient 3	Patient 4	Patient 5	Patient 6	Patient 7	Mean (*SD*)	Effect size *d^1^*
AUDIT	T0	43	11	31	12	13	13	13	19.43 (12.51)	
	T1	34	15	15	15	16	10	20	17.86 (7.69)	.19
	T2	24	15	15	15	16	10	13	15.43 (4.28)	*.57*
	T3	23	15	15	15	17		19	17.33 (3.20)	.40
% reduction on drinking-behavior	T1-T2	29	0	0	0	0	0	35	13	
	T1-T3	32	0	0	0	6 (increase)	n/a	5	3	
BDI-II	T0	37	21	17	22	25	38	17	25.29 (8.81)	
	T1	29	24	15	23	34	32	18	25.00 (7.07)	.45
	T2	17	7	11	12	26	29	10	16.00 (8.45)	1.86***
	T3	24	5	11	17	18		15	15.00 (6.48)	1.29**
% reduction on BDI-II score	T1-T2	41	71	27	48	24	9	44	54	
	T1-T3	17	79	27	26	47	n/a	17	57	
MCQ-30	T0	66	75	53	57	52	77	54	62.00 (10.65)	
	T1	62	74	49	72	63	67	59	63.71 (8.44)	.19
	T2	54	60	58	41	52	61	47	53.29 (7.30)	.88*
	T3	59	54	51	48	56		54	53.67 (3.83)	.93*
PAMS/NAMS	T0	44	40	48	46	48	39	40	43.57 (3.91)	
	T1	42	44	44	28	51	45	42	42.29 (6.99)	.16
	T2	37	35	45	24	46	41	39	38.14 (7.40)	1.40**
	T3	36	56	49	26	46		46	43.17 (10.59)	.19
DTQ	T0	20	32	24	13	23	15	26	21.86 (6.52)	
	T1	23	27	26	12	23	19	23	21.86 (5.05)	.00
	T2	18	17	25	11	21	17	16	17.86 (4.34)	1.16**
	T3	17	15	17	12	19		19	16.50 (2.66)	1.38**

^1^ Cohens d was reported for T0-T1 (T1), T1-T2 (T2) and T1-T3 (T3); AUDIT, Alcohol Use Disorder Identification Test; BDI-II, Beck Depression Inventory II; MCQ-30, Metacognitions Questionnaire 30; PAMS/NAMS, Positive and Negative Alcohol Metacognitions Scale; DTQ, Desired Thinking Questionnaire; *significant on the.05 level; **significant on the.01 level; ***significant on the.001 level.Italic value means that there is a trend significance for this result.

Regarding desire thinking/craving (DTQ), there was no change occurring during the control period (T0-T1), *t*(6) = 0.00, *p* = .50. There was a significant 18% decrease from baseline to post-treatment, *t*(6) = 3.06, *p* ≤ 0.01, *d* = 1.16. Furthermore, desire thinking/craving significantly decreased about 25% three months after treatment termination, *t*(5) = 3.39, *p* ≤ 0.01, *d* = 1.38, demonstrating that further gains in symptom improvement were achieved during the follow-up period.

With respect to depressive symptoms (BDI-II), there was no change occurring during the control period (T0-T1), *t*(6) = 0.13, *p* = .45. Interestingly, there was a significant 54% decrease in depression scores for the group as a whole from pre-treatment to one week post-treatment, *t*(6) = 4.93, *p* ≤ 0.001, *d* = 1.86. Moreover, there was a 57% reduction in depressive symptoms *t*(5) = 3.16, *p* ≤ 0.01, *d* = 1.29 from pre-treatment to three months after treatment termination, demonstrating that further gains were made during the follow-up period. All patients displayed reductions in BDI-II scores at T2 and T3 in comparison to pre-treatment scores.

### 3.2 Changes in metacognitions

Regarding positive and negative alcohol related metacognitions (PAMS/NAMS), no changes were observed during the control period (T0-T1), *t*(6) = 0.42, *p* = .35, *d* = 0.16 for the group as a whole. Thereinafter, there was a significant 10% decrease in alcohol related metacognitive assumptions from pre-treatment to post-treatment, *t*(6) = 3.69, *p* ≤.01, *d* = 1.40. However, no significant changes regarding alcohol related metacognitions could be observed from pre-treatment to follow-up (T1-T3), *t*(5) = -0.47, *p* = .33, *d* = .19, indicating that gains could not be maintained at follow-up.

Looking at metacognitive beliefs (MCQ-30), no changes were observed during the control period, *t*(6) = -0.51, *p* = .32 (T0-T1). Thereinafter, there was a significant 16% reduction in dysfunctional metacognitive beliefs one week after treatment termination, *t*(6) = 2.33, *p* ≤ 0.05, *d* = .88, a 16% decrease three month after treatment termination, *t*(5) = 2.28, *p* ≤ 0.05, *d* = .93, signifying that gains were maintained during the follow-up period.

### 3.3 Feasibility and acceptability

At the initial screening, 12 patients were evaluated, with 10 patients meeting inclusion criteria. After the diagnostic assessment, three patients did not complete treatment: One patient dropped out after the diagnostic appointment, due to long-term rehabilitation placement (female patient, age 60–65, high school diploma, has been abstinent for 3 months prior to the diagnostic appointment, multiple inpatient stays due to complex alcohol withdrawal). One patient could not be contacted after the diagnostic interview (male patient, age 55–60, high school diploma, was applying for reduced earning capacity pension at the time of the study, comorbid depressive disorder, alcohol consumption once a week 4–6 beverages). Lastly, one patient dropped out after two group sessions and could not be contacted (male patient, age 55–60, completed apprenticeship, has been abstinent since 4 weeks prior to study participation, comorbid social anxiety disorder including a fear of groups, multiple inpatient stays due to complex withdrawal as well as multiple rehabilitation stays due to AUD). The remaining seven patients completed at least six out of eight therapeutic sessions (i.e., 75%). Six patients completed questionnaires for all assessment time-points. However, one patient did not complete the follow-up assessment (patient 6, T3) and could not be contacted.

**Figure 1:**
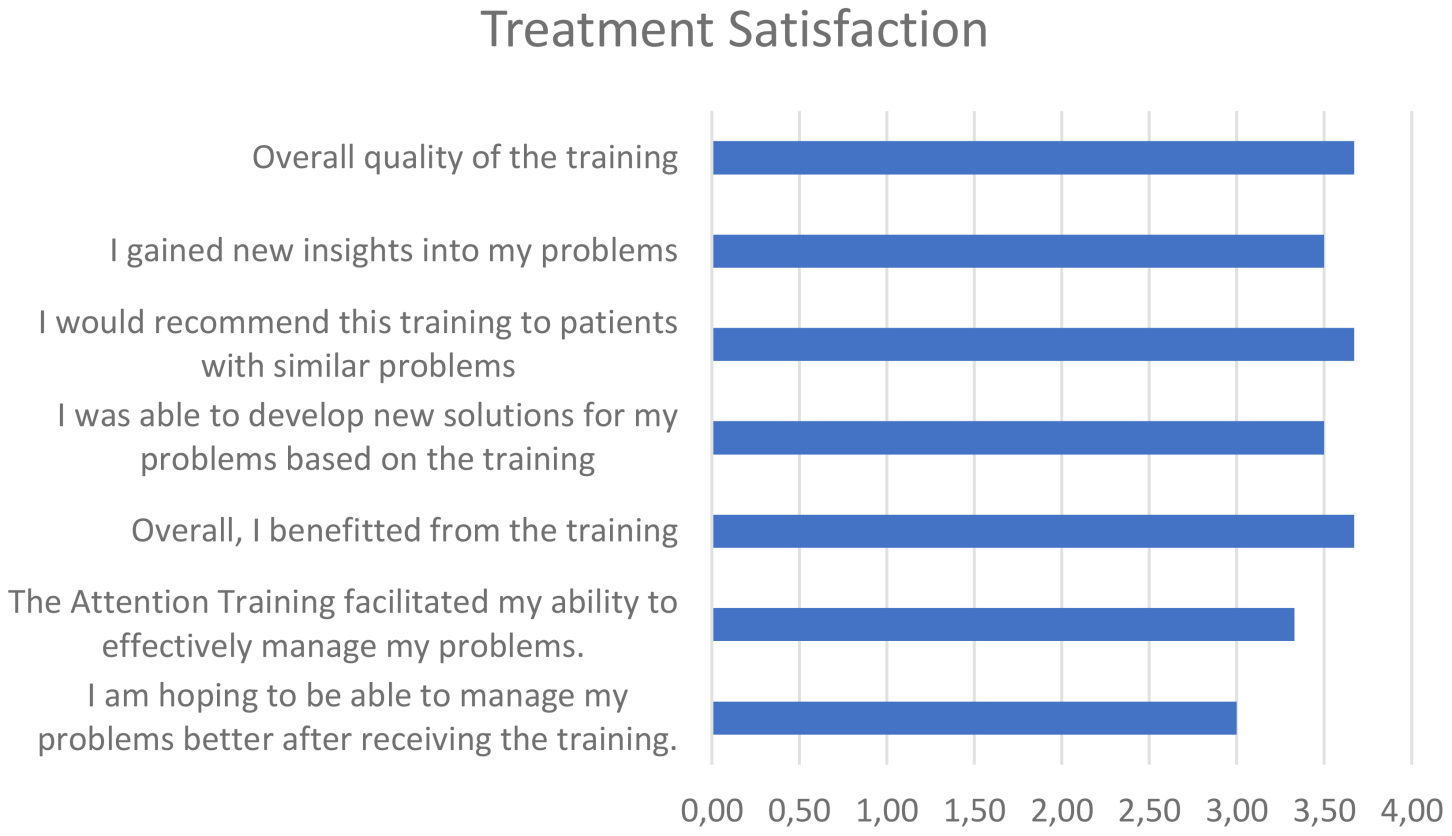
Treatment Satisfaction.

On average, patients were highly satisfied with the group treatment (*M* = 3.39, *SD* = 0.27). Detailed responses to several items included within the patient satisfaction questionnaire can be found in **Figure 1**.

## 4 Discussion

The aim of the study was to examine the efficacy and feasibility of a brief group based MCT intervention for individuals who have been diagnosed with AUD. The results of the current study demonstrate that the implemented intervention was very well accepted amongst the included patients. Solely one patient dropped out of treatment due to unknown reasons. The remaining seven patients completed the group treatment. Furthermore, all patients reported that they were highly satisfied with the treatment and benefitted from the group program.

Overall, our results provide initial evidence for the efficacy of the implemented group based MCT intervention for patients diagnosed with AUD. Specifically, there was a significant and lasting improvement in depressive symptoms (BDI-II) across all included patients displaying high effect sizes. Furthermore, dysfunctional metacognitive beliefs (MCQ-30) improved significantly, with large effect sizes at post-treatment as well as three months after treatment termination. This finding indicates that the implemented MCT was efficient in reducing dysfunctional metacognitive beliefs for an extended period. Aligning with the previous results, desire thinking/craving (DTQ) improved up to three months post intervention, with large effect sizes at both post-treatment measurement time-points. This finding is in turn aligning with Spada et al. (39) triphasic model, which proposes an increase in desire thinking after the activation of positive, alcohol related metacognitive beliefs. As there is a positive association between metacognitive beliefs and desire thinking, it seems comprehensible that desire thinking decreases within the investigated population, as a result of decreased positive metacognitive beliefs.

The results of the present study further align with prior research on group based MCT, which demonstrated notable improvements in metacognitive beliefs and depressive symptoms following six sessions of group-based MCT in a sample of Muslim women diagnosed with Substance Use Disorder (SUD) who currently participate in methadone maintenance therapy (67). Furthermore, a study by Thorslund et al. (68) showed that symptoms of depression and anxiety, as well as metacognitive beliefs significantly improved in a sample of adolescents diagnosed with depressive and anxiety disorders, following six sessions of group-based MCT. However, to date, there is a lack of clinical research on group-based MCT for AUD. Therefore, further studies are required to assess its effectiveness.

Interestingly, there were no changes regarding alcohol abuse symptomatology (AUDIT) across the investigated group. As can be seen within the AUDIT, three patients (43% of the total sample) did not consume alcohol during the entire assessment period (i.e., T0-T3). Therefore, no further improvement can be expected. Furthermore, the lack of improvement regarding the overall alcohol abuse symptomatology could be due to the controlled drinking objective of the study. Specifically, the alcohol consumption of patients who did drink alcohol (patients 1, 5, and 7) decreased in either the amount or the times that alcohol was consumed at post-treatment and follow-up, indicating that the goal of a controlled drinking perspective was achieved. Therefore, when aiming for a controlled drinking objective, future research could investigate the times and the amounts of alcohol consumed during those times, rather than investigating the full spectrum of alcohol abuse disorder as assessed by the AUDIT. On a similar note, scores on AUDIT and PAMS/NAMS worsened from T2 to T3 on average. In order to prevent symptom deterioration, future studies could include booster sessions or increase the duration between treatment sessions towards the end of the treatment for relapse prevention purposes. The presented results on alcohol abuse symptomatology are partially comparable to previous research on several forms of group therapy (e.g., cognitive behavioral therapy) for SUDs, including cocaine, alcohol, and polysubstance use. According to the results of a meta-analysis conducted by Lo Coco et al. (69), there was no change regarding SUD symptomatology across group treatments. Nevertheless, the aforementioned meta-analysis also indicated no changes regarding substance use frequency, which contradicts the findings of the current study. Therefore, the implementation on MCT based group therapy for AUD might be a favorable approach for achieving a controlled drinking objective. However, it is crucial to assess the effectiveness of this treatment technique within future randomized controlled trials.

Taken together, the implemented treatment appears to be feasible and successful in treating symptoms associated with AUD. None of the included patients reported any worsening of their symptoms in comparison to pre-treatment scores. Moreover, patients experienced a decline in various clinical symptoms at post-treatment as well as three months after the intervention. Furthermore, the conducted intervention was highly accepted by all patients.

### 4.1 Limitations

First, the present study included a sample of severely ill clinical patients. These patients had high comorbidities of psychiatric disorders, such as past drug dependence, post-traumatic stress disorder, or depressive disorders. Additionally, most patients were addicted to alcohol for more than 10 years, including several previous inpatient hospitalizations, mostly due to complex withdrawal. Therefore, it remains unclear whether the implemented treatment would yield distinct results for patients with less chronic symptomatology. To test this assumption, future research could conduct statistical analysis for chronic vs. non-chronic alcohol dependent patients to investigate possible diverging outcomes. Second, the included sample consisted of patients between the age of 40 and 60. Therefore, the presented results cannot be transferred to a younger population with a less extensive history of alcohol abuse. Third, due to the high chronicity of alcohol abuse disorder within the presented sample, the included patients might need an extended period of therapeutic assistance to achieve long-term symptom improvement. For example, instead of conducting two therapeutic sessions per week, sessions could take place every second week at the beginning of treatment, and could be extended to every four weeks towards the end of treatment. Fourth, solely one measurement assessing symptoms of depression (i.e., BDI-II) was utilized to assess aspects of negative affectivity. However, previous studies have also demonstrated a correlation between AUD and feelings of anxiety (e.g., 6). Therefore, future research endeavors may consider incorporating measures of anxiety to gain a more thorough understanding of the underlying emotional dysfunctions and corresponding treatment outcomes. Fifth, the case series with an A-B design used for this study can quickly assess the effects of an experimental variable, however, the disadvantage is the inability of this design to distinguish the effect of the intervention from the possible confounds that might occur with the change condition. Lastly, the sample size of the current study was small, and no active or passive control group was included, as the aim of the present study was to test the efficacy and feasibility of the newly invented MCT group treatment. Henceforth, the generalizability of the presented findings needs to be considered with caution. To overcome this drawback, future studies could extend onto the presented findings by conducting a large-scale randomized controlled trial (RCT) to evaluate the effectiveness of the developed MCT group treatment.

## Data availability statement

The datasets presented in this article are available from the corresponding author upon reasonable request. Requests to access the datasets should be directed to JK, julia.kroener@uni-ulm.de.

## Ethics statement

The studies involving humans were approved by ethics committee of the State Physician Chamber of Baden-Wuerttemberg. The studies were conducted in accordance with the local legislation and institutional requirements. The participants provided their written informed consent to participate in this study. Written informed consent was obtained from the individual(s) for the publication of any potentially identifiable images or data included in this article.

## Author contributions

JK: Conceptualization, Data curation, Formal analysis, Methodology, Project administration, Supervision, Visualization, Writing – original draft, Writing – review & editing. ME: Conceptualization, Data curation, Investigation, Methodology, Writing – original draft. ZS-V: Conceptualization, Funding acquisition, Methodology, Project administration, Resources, Supervision, Writing – review & editing.
